# Oral implanomics: transitioning from experience-based discipline to AI-driven therapeutic paradigm – radiomics and artificial intelligence in oral implantology

**DOI:** 10.3389/froh.2026.1895266

**Published:** 2026-06-26

**Authors:** Hongyang Ma

**Affiliations:** 1 Second Affliated Hospital, Harbin Medical University, Harbin, China; 2 School of Stomatology, Harbin Medical University, Harbin, China; 3 Peking University Hospital of Stomatology, Beijing, China

**Keywords:** artificial intelligence, cone-beam computed tomography, machine learning, oral implantology, precision dentistry, radiomics

## Abstract

Oral implantology has experienced substantial digital transformation over the past two decades. The adoption of cone-beam computed tomography (CBCT), computer-assisted surgery, and artificial intelligence (AI) has improved diagnostic accuracy, treatment planning, and procedural precision. However, clinical decision-making remains largely dependent on conventional radiographic interpretation and clinician experience. We propose the concept of Implanomics (Implant-Omics) as an integrative framework combining radiomics, AI, and digital workflows. Radiomics, which converts medical images into quantitative data, offers new opportunities to extract imaging biomarkers from routine CBCT examinations. By characterizing bone morphology, trabecular architecture, and image texture beyond visual assessment, radiomics may support more objective evaluation of implant sites and improve prediction of treatment outcomes. When combined with machine-learning algorithms, these imaging-derived features can be incorporated into predictive models for risk assessment, implant planning, and longitudinal monitoring. In this Perspective, we discuss the evolution of implant dentistry from experience-based planning toward imaging-driven decision support and examine the emerging role of radiomics and AI in precision implant care. We highlight current applications, key challenges, and future directions for integrating quantitative imaging analysis into clinical workflows. Although substantial validation and implementation challenges remain, the convergence of radiomics, artificial intelligence, and digital implant technologies may contribute to more individualized and evidence-informed treatment strategies in implant dentistry.

## 1 Reconsidering the foundations of clinical decision-making in implant dentistry

Dental implant therapy is widely regarded as a predictable and effective treatment option for the rehabilitation of missing teeth (1). Long-term survival rates frequently exceed 95% over ten years, supporting its role as a reliable treatment modality (2–4). Nevertheless, the clinical principles guiding implant treatment planning remain largely experience-based and rely heavily on anatomical assessment.

Current treatment planning typically involves interpretation of radiographic imaging, assessment of local anatomical conditions, and evaluation of patient-related risk factors (5). Although digital planning software and computer-assisted surgical techniques have improved procedural accuracy and reproducibility (6, 7), the underlying rationale for many clinical decisions continues to depend on expert judgment and accumulated experience (8).

These challenges become increasingly relevant as implant dentistry expands into more complex treatment scenarios (9). The growing availability of digital technologies, large-scale clinical datasets, and advanced computational methods therefore raises an important question: can implant treatment planning move beyond descriptive anatomical evaluation toward a more predictive and biologically informed model of care (10, 11)?

## 2 Digital transformation and the current limits of data utilization

Over the past two decades, digital technologies have progressively reshaped many aspects of implant dentistry (12). The adoption of CBCT, intraoral scanning, computer-aided design and manufacturing (CAD/CAM), and computer-assisted surgical systems has improved diagnostic capabilities, treatment planning efficiency, and procedural accuracy (13). These developments have contributed to increasingly predictable workflows and have enhanced communication among clinicians, technicians, and patients (14).

More recently, AI has emerged as an additional layer within digital implant workflows (15). Applications have been reported across a broad range of tasks, including automated anatomical segmentation, radiographic interpretation, implant identification, virtual treatment planning, prosthetic design, and outcome prediction (16). Deep-learning algorithms have demonstrated high performance in identifying relevant anatomical landmarks, assessing alveolar bone dimensions, and processing CBCT datasets with a level of consistency that may reduce operator-dependent variability (17). Similar approaches have also been applied to automated implant recognition and preliminary treatment planning, often achieving performance comparable to that reported for experienced clinicians under controlled experimental conditions (18, 19).

The integration of AI into diagnostic imaging has attracted particular attention (20). Automated segmentation systems can rapidly identify structures such as the mandibular canal, maxillary sinus, adjacent roots, and edentulous regions, thereby reducing the time required for image analysis and potentially improving reproducibility (21). In parallel, machine-learning models have been developed to estimate implant-related outcomes, including survival probability, peri-implant bone loss, and the risk of biological or mechanical complications (22). Although the predictive accuracy reported by many studies is encouraging, most currently available models remain limited by retrospective study designs, relatively small datasets, and restricted external validation (23).

Advances have also been reported in surgical execution. Dynamic navigation systems, robotic assistance, and real-time tracking technologies have improved the transfer of virtual treatment plans to the clinical setting (24). Experimental and clinical investigations suggest that these technologies may reduce deviations between planned and actual implant positions while simultaneously enhancing procedural standardization. Furthermore, educational studies indicate that AI-supported planning tools and navigation systems may facilitate skill acquisition among less experienced practitioners by providing objective guidance and immediate feedback during training (25).

Despite these important achievements, digital transformation has not fundamentally altered the nature of the information that underpins clinical decision-making. Most contemporary digital workflows remain largely dependent on anatomical imaging and conventional clinical variables. AI systems typically learn associations between radiographic features and treatment outcomes, but they generally lack direct access to the molecular and biological mechanisms that influence osseointegration, peri-implant tissue stability, and long-term implant performance. Consequently, many predictive models function primarily as sophisticated pattern-recognition systems rather than as mechanistic representations of host–implant interactions.

This distinction has important implications. A model capable of accurately predicting implant failure from imaging data may assist clinical decision-making, yet its predictions do not necessarily explain the biological pathways responsible for an adverse outcome. Factors such as inflammatory susceptibility, genetic predisposition, immune regulation, microbial ecology, and tissue regenerative capacity remain only partially represented within conventional imaging datasets. As a result, AI-generated predictions may identify patients at increased risk without fully clarifying the underlying biological basis of that risk (26).

The current generation of digital technologies should therefore be viewed as an important step toward data-driven implant dentistry rather than as its final stage. Digital imaging, computer-assisted surgery, and machine-learning algorithms have substantially improved the acquisition, organization, and interpretation of clinical information. However, achieving a more individualized understanding of treatment response will likely require the incorporation of biological data that extend beyond radiographic phenotypes (27). In this context, omics technologies provide a promising opportunity to complement existing digital workflows with molecular information capable of characterizing patient-specific biological conditions at a much greater level of detail (28).

## 3 Radiomics: extracting quantitative information from dental imaging

**Figure 1:**
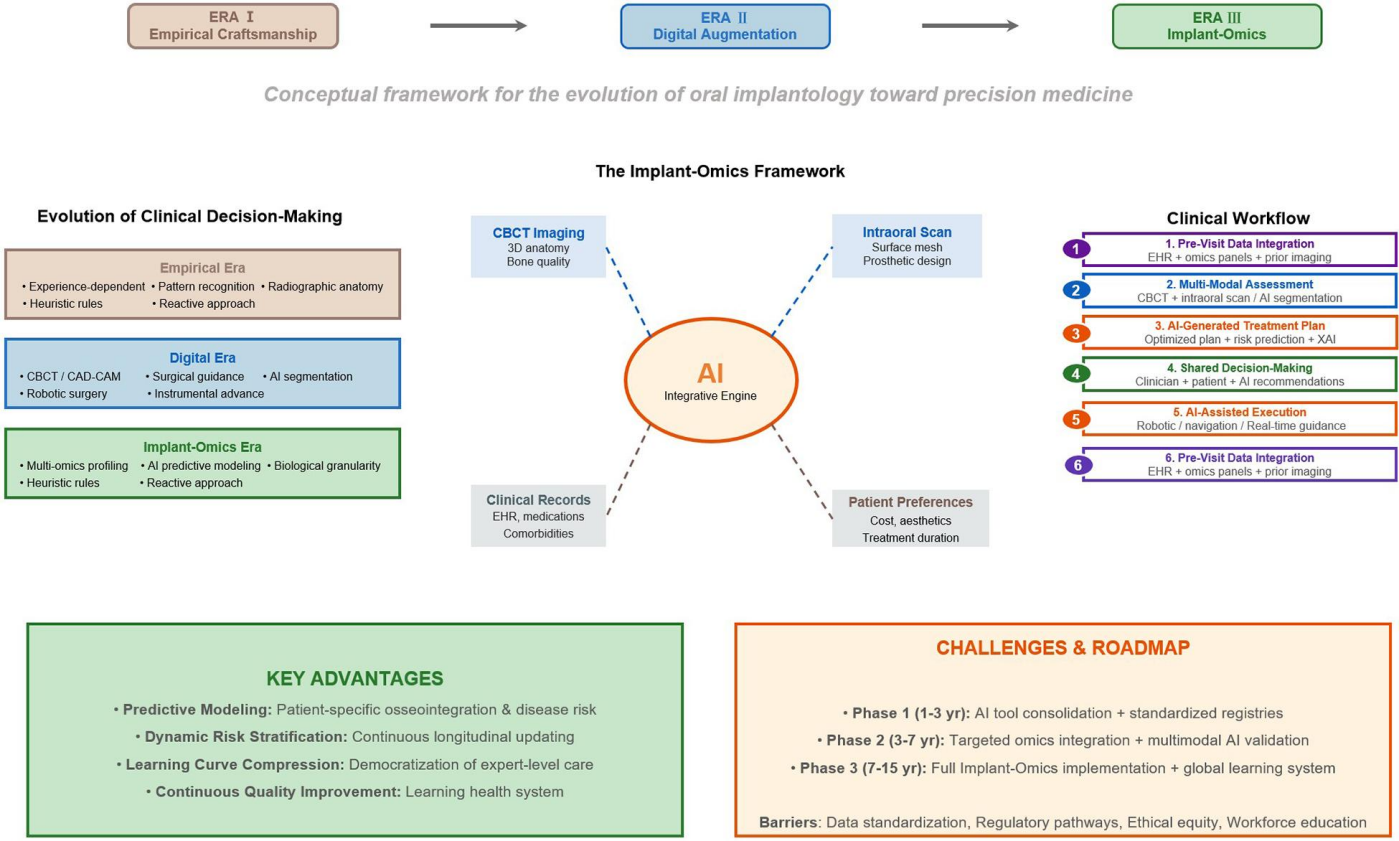
Conceptual framework of the Implant-Omics (Implanomics) paradigm.

Radiomics refers to the high-throughput extraction of quantitative features from medical images (29–31). As detailed in Section 4.1, this approach can be applied to routine CBCT scans to derive imaging biomarkers that may support bone quality assessment and treatment planning (Figure 1). A brief overview is provided here, while a full discussion of quantitative imaging biomarkers is presented in Section 4.1.

## 4 Imaging-based precision implant dentistry

The increasing availability of high-resolution dental imaging and advanced computational methods is creating new opportunities for data-driven implant care (32). Traditionally, implant treatment planning has relied on visual interpretation of radiographic images, supplemented by clinical examination and practitioner experience. Although these approaches remain fundamental to clinical practice, they may not fully utilize the quantitative information contained within modern imaging datasets.

Radiomics and AI offer a potential framework for transforming routine imaging examinations into sources of objective and clinically actionable information (33). By extracting quantitative imaging biomarkers and integrating them with predictive algorithms, these technologies may support more individualized assessment of anatomical conditions, treatment-related risks, and long-term outcomes. Within this context, imaging-based precision implant dentistry can be viewed as an emerging model in which clinical decision-making is increasingly informed by quantitative image analysis and computational prediction rather than solely by subjective interpretation (34).

### 4.1 Quantitative imaging biomarkers

Conventional implant planning primarily relies on measurements of bone height, width, and anatomical relationships obtained from CBCT examinations. While these parameters provide essential information for treatment planning, they represent only a fraction of the data available within three-dimensional imaging datasets.

Radiomics is an emerging approach that converts medical images into mineable, high-dimensional data. In implant dentistry, cone-beam computed tomography (CBCT) provides three-dimensional imaging datasets from which quantitative features can be extracted, including first-order intensity statistics, shape descriptors, texture patterns (e.g., gray-level co-occurrence matrices), and higher-order wavelet parameters. These features reflect underlying bone microstructure, trabecular architecture, and local heterogeneity beyond what is visible to the human eye (35). These features may include image intensity distributions, shape descriptors, texture patterns, and higher-order statistical parameters that reflect structural heterogeneity within the alveolar bone. Such imaging biomarkers have the potential to provide additional information regarding bone quality, trabecular organization, cortical integrity, and local anatomical complexity (36).

In implant dentistry, quantitative imaging biomarkers may contribute to several clinically relevant applications. These include assessment of implant site suitability, evaluation of bone quality, prediction of primary implant stability, and estimation of peri-implant bone remodeling potential. Furthermore, radiomic features may facilitate objective comparison of implant sites and support standardized treatment planning across clinicians and institutions (37).

Although many proposed imaging biomarkers remain under investigation, their development represents an important step toward transforming radiographic examinations from descriptive imaging tools into quantitative sources of prognostic information.

### 4.2 AI-assisted risk prediction

Risk assessment remains one of the most challenging aspects of implant treatment planning. Current clinical decisions often rely on established risk factors, including bone quantity, systemic health status, smoking habits, and previous periodontal disease. While these factors provide valuable guidance, they may not fully capture the complexity of interactions influencing implant outcomes (38).

Artificial intelligence offers an opportunity to improve risk prediction through analysis of large and multidimensional datasets (39). Machine-learning algorithms can identify patterns within imaging data that may not be readily detectable by human observers and integrate these findings into predictive models (40). Recent studies have demonstrated the feasibility of AI-based systems for predicting implant survival, peri-implant bone loss, immediate implant placement feasibility, and potential surgical complications using preoperative imaging information (41).

The integration of radiomic features into predictive algorithms may further enhance model performance by providing quantitative descriptors of local anatomical conditions. Rather than generating simple binary outcomes, future AI systems may provide individualized risk estimates, confidence intervals, and probability-based predictions that assist clinicians in selecting appropriate treatment strategies.

Importantly, AI-assisted prediction should be viewed as a complementary tool rather than a replacement for clinical judgment. Predictive models are most valuable when used to support clinical reasoning, improve risk communication, and facilitate evidence-informed decision-making.

### 4.3 Dynamic imaging surveillance

Implant treatment does not end at the time of surgical placement. Long-term success depends on continuous monitoring of osseointegration, peri-implant tissue stability, prosthetic function, and maintenance compliance. Conventional follow-up protocols typically rely on periodic clinical examination and radiographic evaluation, with disease detection occurring after structural changes become visible (42).

Advances in quantitative imaging analysis may enable a more proactive approach to postoperative surveillance. Sequential radiographic examinations can be analyzed using automated algorithms capable of detecting subtle alterations in bone density, trabecular architecture, and marginal bone levels before these changes become clinically apparent (43). Such approaches may facilitate earlier identification of unfavorable biological responses and allow timely intervention.

Future surveillance systems may combine serial imaging data with AI-based analytical models to continuously update patient-specific risk profiles. By monitoring temporal changes rather than isolated observations, these systems could provide a more comprehensive understanding of implant performance throughout the treatment lifecycle (44).

Although significant validation is still required, dynamic imaging surveillance represents a promising application of precision implant dentistry and may contribute to more individualized maintenance strategies in the future.

### 4.4 Explainable AI for clinical decision support

For AI technologies to achieve widespread clinical acceptance, predictive accuracy alone is insufficient. Clinicians must be able to understand, evaluate, and critically interpret algorithm-generated recommendations before incorporating them into patient care.

Explainable artificial intelligence (XAI) aims to address this challenge by providing transparent insight into the factors influencing model predictions (45). Rather than functioning as opaque “black-box” systems, explainable models identify the imaging features and variables that contribute most strongly to a particular recommendation or risk estimate.

In implant dentistry, explainable AI may allow clinicians to understand why a system recommends a specific implant position, identifies a site as high risk, or predicts increased probability of peri-implant bone loss. Such transparency improves trust, facilitates communication with patients, and supports informed clinical decision-making (46).

Equally important, explainability enables clinicians to recognize potential limitations and biases within algorithmic outputs. Human oversight remains essential for integrating computational recommendations with patient preferences, clinical context, and professional experience (47). Consequently, the future role of AI in implant dentistry is likely to be collaborative rather than autonomous, combining the analytical capabilities of computational systems with the judgment and responsibility of trained clinicians.

## 5 Artificial intelligence as an integrative analytical platform

The clinical value of the Implant-Omics (Implanomics) framework ultimately depends on the ability to integrate diverse forms of information into actionable knowledge. Imaging datasets, radiomic features, clinical records, behavioral variables, and longitudinal outcome measurements each provide important but incomplete perspectives on patient status. Interpreting these data streams simultaneously exceeds the capacity of conventional analytical approaches and presents a major challenge for the implementation of precision-oriented implant care.

Artificial intelligence offers a potential solution to this challenge by providing computational methods capable of identifying patterns, relationships, and predictive signals within large and heterogeneous datasets. Rather than serving as an independent clinical authority, AI may function as an analytical platform that facilitates the translation of complex biological information into clinically meaningful insights. Within the Implant-Omics framework, its principal role is not to replace clinical reasoning but to support the integration, interpretation, and application of multidimensional patient data.

## 6 Challenges and future directions

Despite the rapid development of radiomics and AI, several challenges must be addressed before these technologies can be routinely integrated into implant dentistry. Future implementation will depend not only on technological progress but also on data quality, clinical validation, and professional acceptance.

### 6.1 Data standardization and clinical validation

The reliability of radiomics and AI-based prediction models depends heavily on the quality and consistency of imaging data. Variations in CBCT acquisition protocols, scanner characteristics, image reconstruction parameters, and segmentation methods may influence extracted radiomic features and limit reproducibility across institutions. In addition, most current studies rely on retrospective datasets and relatively limited sample sizes. Future research should prioritize multicenter collaborations, standardized imaging protocols, and prospective validation studies to establish robust and generalizable predictive models suitable for clinical application.

### 6.2 Explainability, ethics, and regulatory considerations

For AI-assisted decision-support systems to gain widespread clinical acceptance, predictive performance alone is insufficient. Clinicians must be able to understand and evaluate the factors underlying algorithmic recommendations. Explainable AI approaches may improve transparency and facilitate trust in model outputs. At the same time, issues related to patient privacy, data security, algorithmic bias, and legal accountability require careful consideration. Appropriate regulatory frameworks and continuous performance monitoring will be essential to ensure safe and responsible implementation.

### 6.3 Future perspectives for imaging-driven implant dentistry

Despite these challenges, the integration of radiomics and AI offers a promising pathway toward more objective and individualized implant care. Quantitative imaging biomarkers derived from routine CBCT examinations may support assessment of implant site characteristics, prediction of treatment outcomes, and longitudinal monitoring of peri-implant conditions. Combined with advanced machine-learning algorithms, future digital workflows may provide clinicians with personalized risk assessment and decision-support tools, contributing to more precise, evidence-informed, and patient-centered implant treatment strategies.

## 7 Conclusions

Despite ongoing challenges related to validation standards, data harmonization, transparency, and regulatory oversight, the convergence of radiomics, artificial intelligence, and digital implant workflows holds significant promise for advancing imaging-driven precision implant dentistry. Future research should prioritize translating quantitative imaging biomarkers into practical clinical tools, guided not only by technological innovation but also by rigorous scientific validation and patient-centered care—with the ultimate goal of generating clinically meaningful knowledge to improve treatment decisions and patient outcomes. Whether “Implant-Omics” will become an established component of routine practice remains uncertain; nevertheless, the integration of molecular biology, digital technologies, and computational analytics offers a compelling direction for continued investigation. Such interdisciplinary efforts may foster more individualized, predictive, and evidence-informed approaches to implant therapy, thereby supporting the ongoing evolution of implant dentistry toward a more biologically integrated model of care.

## Data Availability

The original contributions presented in the study are included in the article/Supplementary Material, further inquiries can be directed to the corresponding author.
